# Diagnostic Approach to Adult Erythroderma: A Rare Case of Sezary Syndrome

**DOI:** 10.30699/ijp.2024.2022723.3258

**Published:** 2024-10-02

**Authors:** Quri Meihaerani Savitri, Fitria Rettobyan, Linda Astari, Amira Suryani Rahmatika, Muhammad Noor Diansyah, Putu Niken Ayu Amrita, Pradana Zaky Romadhon, Sipirianus Ugroseno Yudho Bintoro, Ami Ashariati, Merlyna Savitri

**Affiliations:** 1 *Department of Internal Medicine, Faculty of Medicine, Airlangga University, Surabaya, Indonesia*; 2 *Department of Dermatology and Venereology, Faculty of Medicine, Airlangga University, Surabaya, Indonesia*; 3 *Hematology and Medical Oncology Division, Department of Internal Medicine, Dr. Soetomo General Teaching Hospital, Surabaya, Indonesia*; 4 *Department of Internal Medicine, Dr. Soetomo General Teaching Hospital, Surabaya, Indonesia*

**Keywords:** Biopsy, Cutaneous T-cell lymphoma, Immunohistochemistry

## Abstract

**Background & Objective:**

Sezary Syndrome is an uncommon leukemic variant of Cutaneous T-cell Lymphoma (CTCL), comprising only 5% of all CTCL cases. The rarity of this syndrome emphasizes the critical need to comprehend its distinct clinical presentation, diagnosis, and treatment.

**Case Presentation:**

A 51-year-old man was admitted with itchy, persistent, and extensive erythematous patches, ulcers, lumps, lymphadenopathy, alopecia, and nail dystrophy that had been present for eight months. Laboratory findings showed elevated LDH and 𝛽2-microglobulin. Peripheral blood smear analysis confirmed the presence of Sezary cells, while imaging revealed multiple lymph node enlargements. Skin biopsy and immunohistochemistry suggested cutaneous T-cell lymphoma (CTCL), while immunophenotyping verified a diagnosis of Sezary syndrome . The patient underwent fluid therapy, systemic antibiotics, topical antibiotics, phototherapy, and chemotherapy. Tenofovir was given due to the hepatitis B co-infection. Despite the improvement when discharged from the hospital, the patient's health eventually deteriorated, which led to death at home.

**Conclusion:**

This patient presented with Sezary Syndrome, exhibiting atypical dermatologic manifestations that must be differentiated from other causes of erythroderma. This case highlights the importance of a comprehensive diagnostic approach, including clinical evaluation, laboratory tests, imaging, and biopsies. Sezary Syndrome is an inherently aggressive malignancy, characterized by a poor response to treatment and a low 5-year survival rate.

## Introduction

The uncommon leukemic form of cutaneous T-cell lymphoma (CTCL) (5% of all CTCL cases) is Sezary syndrome (SS) (1). This malignancy occurs only in male adults, more commonly in the patients older than 50 years, with reported annual incidences of 0.1 to 0.3 cases per 1 million population (2,3). The mutation from the chronic activation of T-cells by antigen-presenting cells was allegedly the etiology of SS (2). However, the precipitating factors that culminated in the disease are still unknown (4). 

Confirmation for diagnosis of SS is still burdensome since there is no “gold standard” test. The diagnosis is confirmed through a combination of clinical manifestation, histopathologic examination, and immunohistochemical findings (2). SS manifests as an erythrodermic and pruritic form, characterized by peripheral lymphadenopathy and neoplastic T-cells with cerebriform nuclei (Sezary cells) in the skin, lymph nodes, or peripheral blood (5). The diagnosis criteria established by The International Society of Cutaneous Lymphomas (ISCL) consist of an absolute Sezary count ≥1000/uL or an expanded CD4+ cell count with a CD4/CD8 ratio ≥10 or aberrant loss of one or more pan-T-cell antigens (commonly CD7 and/or CD26 in SS cases), or polymerase chain reaction (PCR) detection of clonal T-cell receptor (TCR) gene rearrangement, or cytogenetic demonstration of an abnormal clone (6,7). 

Different treatment approaches for SS have been recommended by the European Organization for Research and Treatment of Cancer (EORTC) (1). Long-term remission is, however, rarely induced by most current therapy. Given the aggressive nature of the disease, the overall 5-year survival rate is 36.2% (5). Here, we present a case of SS with the classic manifestation of erythroderma, generalized lymphadenopathy, and the presence of Sezary cells, with atypical skin manifestation with skin ulcers and scattered skin lumps and aggravating factors which septic shock due to a urinary tract infection and hepatitis B co-infection to provide insights into SS diagnosis and its associated infection treatment. This case report adheres to the SCARE Criteria (8).

## Case Presentation

A 51-year-old man was admitted to the Internal Medicine Ward due to persistent widespread red, scaly, and itchy rashes of eight months duration. The symptoms initially appeared as red patches on his trunk and later spread to his hands, feet, and face. The patient had previously experienced lymph node enlargement on his head that was surgically removed a year ago from a previous hospital. However, post-surgery, the lymph node enlargement multiplied throughout his body and formed scattered skin lumps. The initial suspicion from the previous hospital was leprosy; however, an acid-fast bacilli test yielded negative results. In the recent week, the patient developed a fever, chills, and a notable weight loss of 10 kg over eight months, resulting in pronounced weakness and limited mobility.

**Fig. 1:**
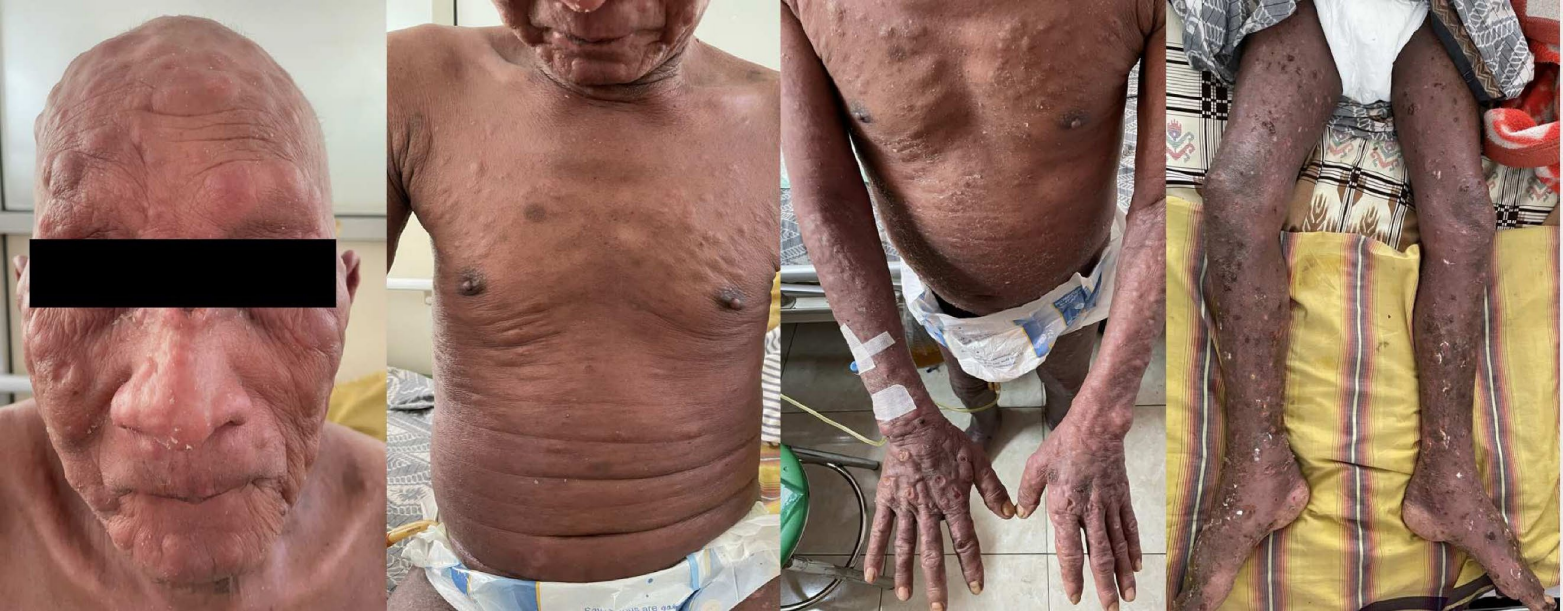
Clinical presentation of the patient consists of multiple nodules and erythematous hyperker- atosis patches all over the face, neck, trunk, and extremities and multiple lymphadenopathy on both neck and inguinal nodes. He also presented with numerous ulcers in his nose, and upper and lower extremities. Madarosis, alopecia, and nail dystrophy were also present.

?Figure 1). Upon abdominal examination, the patient's abdomen was distended with normal bowel sounds. There was shifting dullness on percussion and lower quadrant tenderness on palpation. No palpable masses or organ enlargement were detected. Both of the patient's legs were swollen, exhibiting pitting edema.

**Fig. 2:**
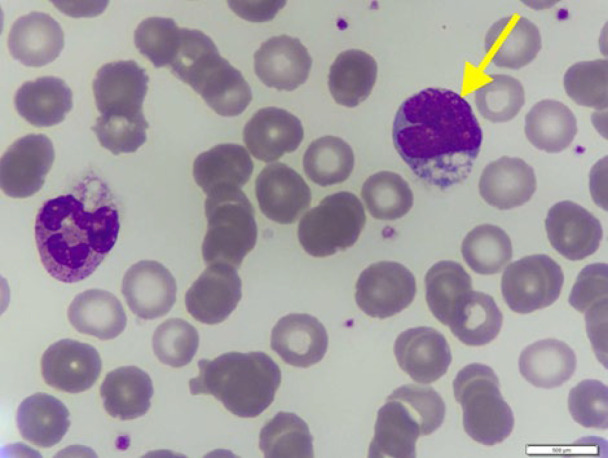
Yellow arrow: Sezary cell found in the patient’s peripheral blood smear.

**Fig. 3:**
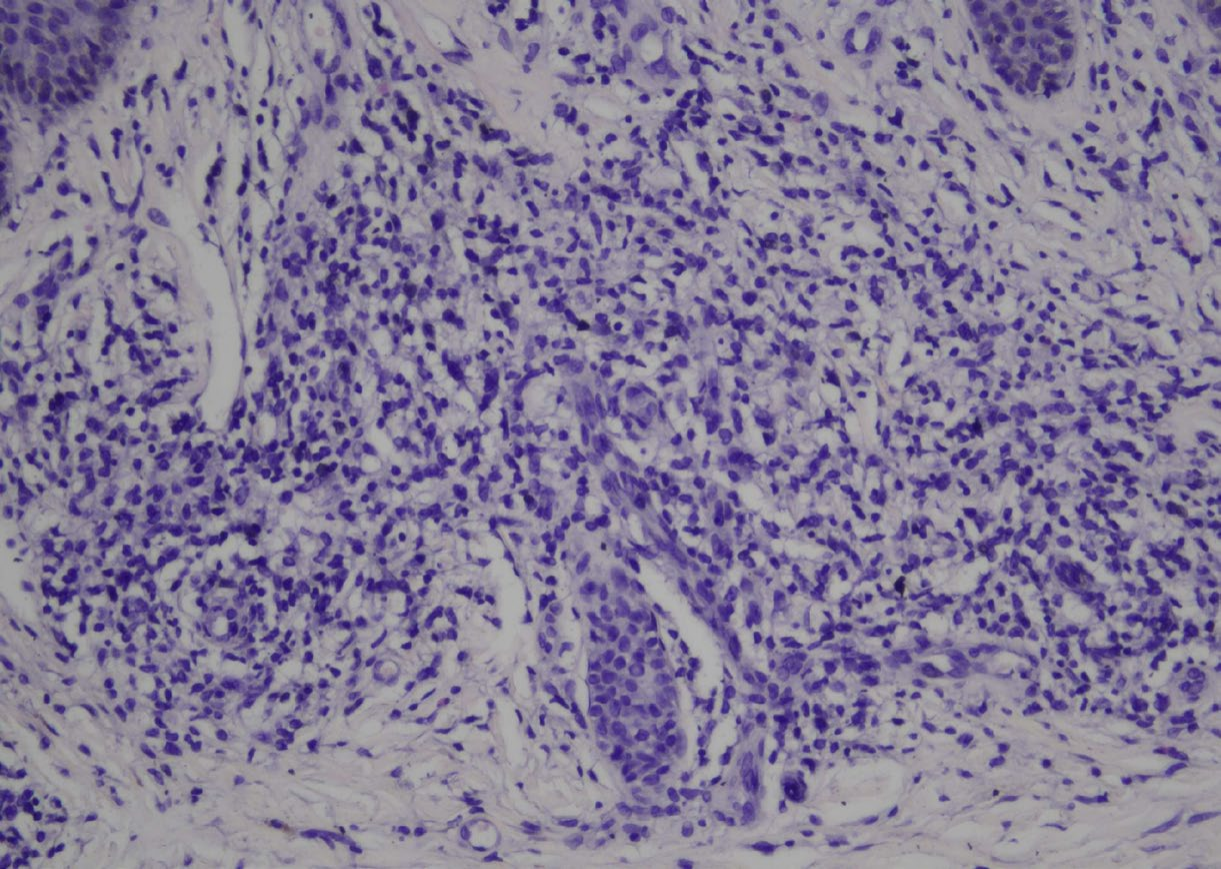
Pathological results of the skin biopsy form the patient showing epidermal acanthosis and parakeratosis with elongated rete ridge. There is also an lymphocytic infiltration along the dermo-epidermal junction and within the superficial dermis. These findings suggested a CTCL.

**Fig. 4:**
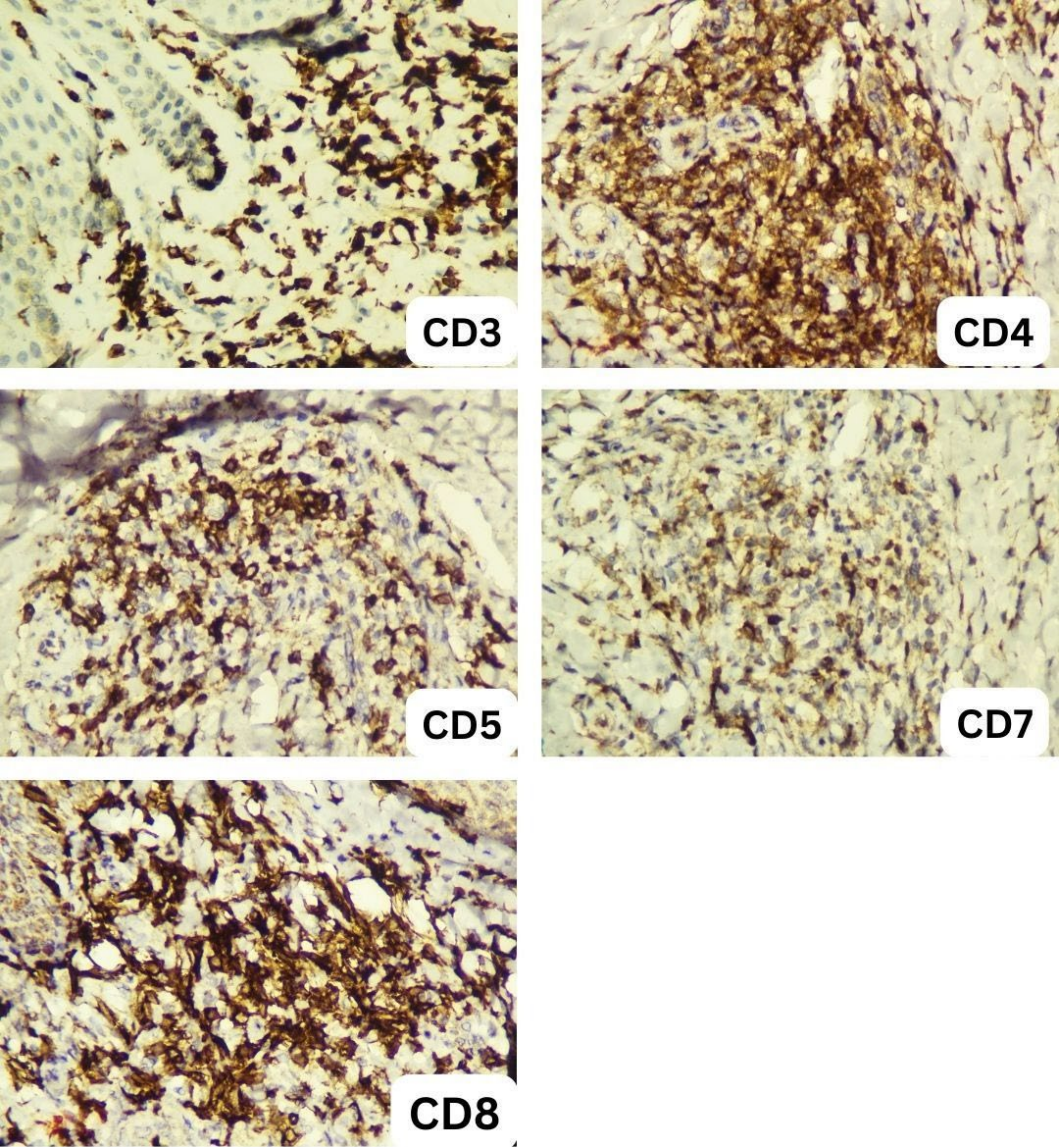
Immunohistochemical result showing positivity for CD3, CD4, CD5, CD7, CD8.

**Fig. 5:**
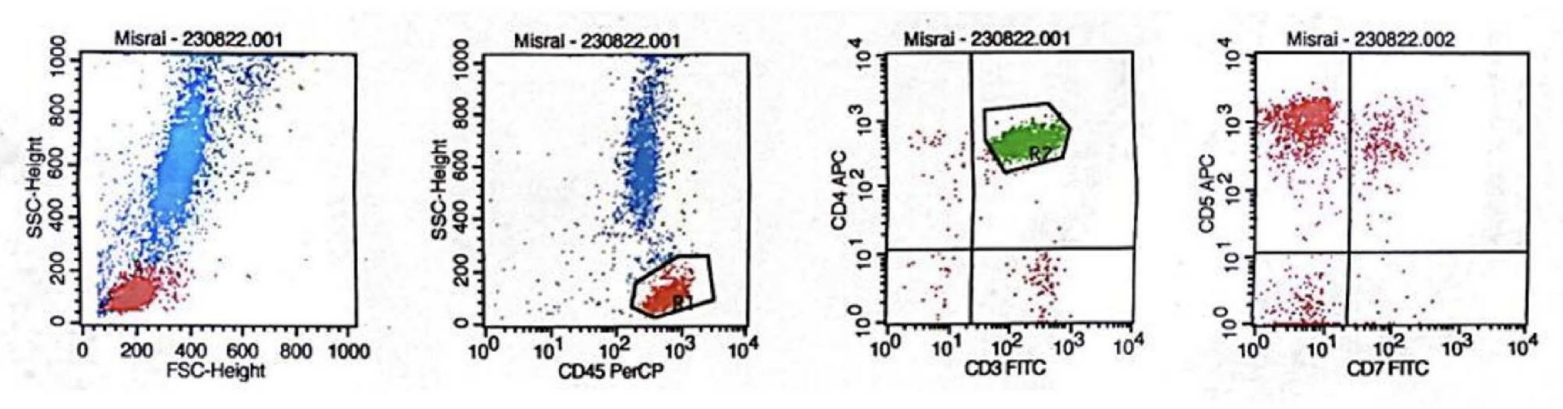
Immunophenotyping results showing an expansion of T-cell CD4+(91 percent of total lymphocyte count), which predominantly is associated with aberrant loss of CD7 (70 percent of total lymphocyte count). SS is an expansion T-cell CD4+ and aberrant loss one or more of the pan T-cell antigen (most common CD7 and/or CD26),

Blood tests revealed several abnormalities, including anemia, an elevated leukocyte count, elevated absolute lymphocyte count, low albumin, high LDH levels, and an increased 𝛽2-microglobulin level. The presence of Sezary cells was confirmed by peripheral blood smear examination (Figure 2), which consists of 9% of the total lymphocytes (1,116 Sezary cells). Ultrasound imaging of lymph nodes identified multiple enlarged nodes on the neck. The skin biopsy displayed epidermal acanthosis and parakeratosis with an elongated rete ridge. Notably, the lymphocytic cell infiltration was noted along the dermo-epidermal junction and within the superficial dermis. These findings suggested a CTCL (Figure 3). Immunohistochemical results showed positivity for CD3, CD4, CD5, CD7, and CD8, indicating cutaneous T-cell lymphoma (CTCL) Sezary Syndrome (Figure 4). Immunophenotyping of the lymphocytes by flow cytometry revealed an expansion of T-cell CD4+ (making up 91% of the total lymphocyte count) and a significant reduction of CD7 (constituting 70% of the total lymphocyte count) (Figure 5). Based on our Pathology Anatomy Department, the flow cytometry results indicated a diagnosis of Sezary Syndrome . The results of appropriate physical examinations and other diagnosis examinations supported the diagnosis. The patient was also diagnosed with chronic hepatitis B with reactive HbsAg, non-reactive HBeAg, and HBV DNA was detected at 7.8×10^6^ IU/mL.

The treatment plan included administration of 500 mL of tutofusin and 1500 mL of normal saline for fluid support. Antibiotics, specifically ciprofloxacin at 400 mg every 12 hours and paracetamol at 1 gram every 8 hours, were part of the regimen. Hepatitis B antiviral, tenofovir at 300 mg every 24 hours. CHOP regimen for chemotherapy was initiated, and topical treatments, such as ointment and moisturizer cream, were applied alongside UVB phototherapy. Despite these efforts, the patient's condition continued to worsen. Additional complications surfaced, including hepatitis B and blood and ulcer cultures identified antibiotic-resistant bacteria. Despite diligent management involving medications, phototherapy, and red blood cell transfusions, the patient's health did not show improvement. Regrettably, he passed away at home two weeks after being discharged from the hospital.

**Fig. 6:**
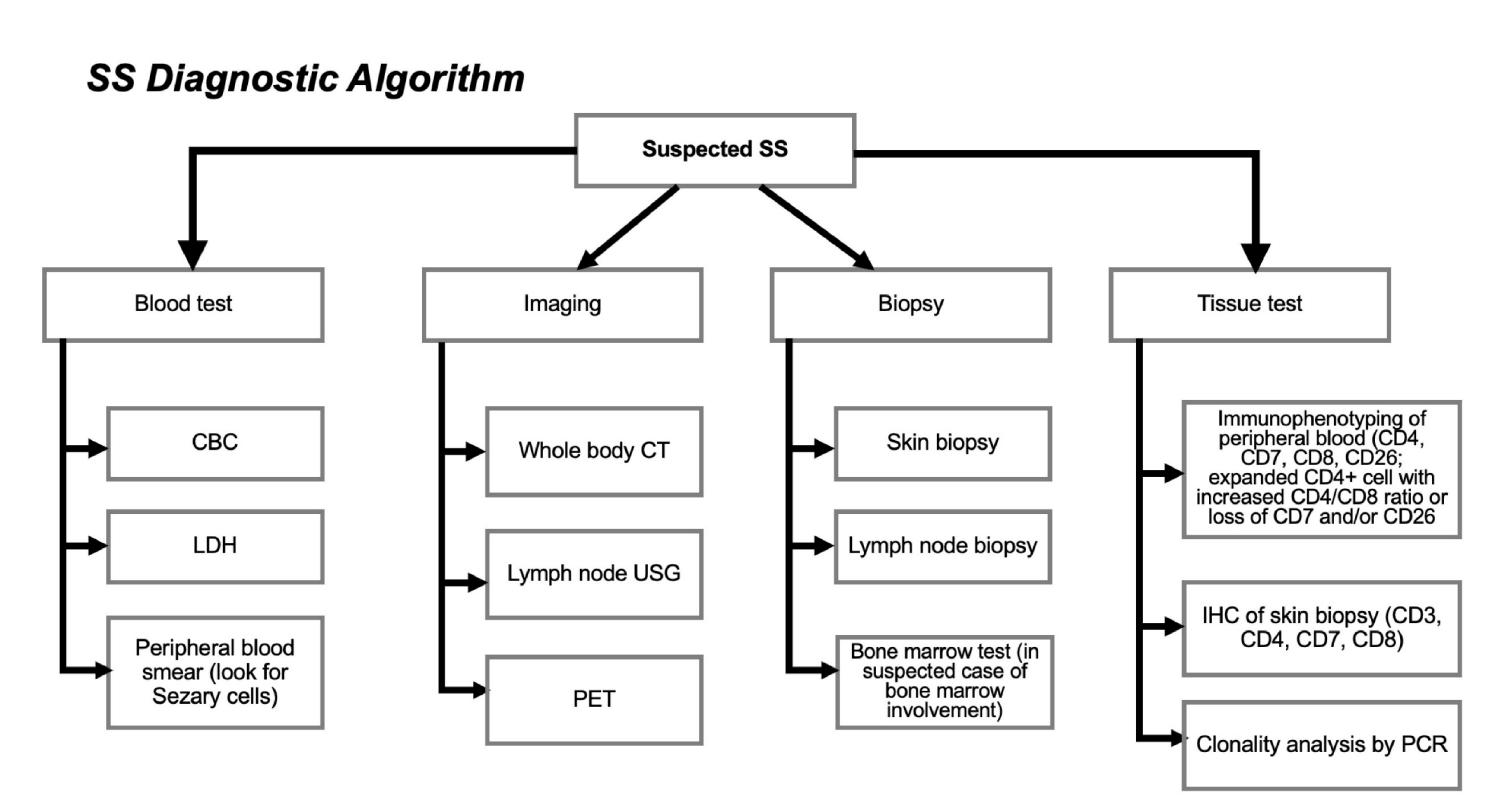
Diagnostic approaches to Sezary syndrome.

## Discussion

Erythroderma is a severe skin condition characterized by widespread redness and peeling ≥90% covering of the body. Psoriasis, spongiotic dermatitis, drug reactions, pityriasis rubra pilaris, and T-cell lymphomas are among the main causes of adulthood. (9). Key distinctions for psoriasis involve nail pitting, face sparing, and scattered pustules. Spongiotic dermatitis is associated with edema disorders like atopic dermatitis. Drug-induced erythroderma is linked to specific medications and typically resolves within weeks after discontinuation. Pityriasis rubra pilaris lesions are frequently well-defined from the unaffected skin, which forms an “island of sparing”. While Sezary cells are not present in mycosis fungoides, they are exclusively seen in SS (9). Sezary Syndrome (SS) primarily affects individuals aged 55-60, displaying erythroderma, lymph node enlargement, and Sezary cells (10–12). Various symptoms such as ectropion, fissuring, and fever may emerge. Blood tests and imaging aid diagnosis. Skin biopsy typically reveals perivascular lymphocytes with CD4+ and CD7- cells. Staging employs the TNMB system (13–15).

This patient met the Sezary Syndrome (SS) criteria, displaying features such as erythroderma, Sezary cells, and lymph node involvement. The diagnosis was confirmed through consistent findings in lymph node ultrasound (USG) and biopsy, which revealed an expansion of CD4+ cells and loss of CD7, placing the patient at stage IVA1. In accordance with EORTC consensus recommendations for treating mycosis fungoides/Sezary syndrome, the treatment approach involved phototherapy (NB-UVB) and chemotherapy (CHOP) (1). NB-UVB was preferred over PUVA due to its safety and effectiveness, avoiding side effects like nausea and headache, as well as having a lower risk of skin cancer than PUVA (1,5,16,17). CHOP (cyclophosphamide, doxorubicin, vincristine, and prednisone) is a common combination chemotherapy regimen for SS with well-known high response rates (70-80%) (6). Unfortunately, the patient's condition deteriorated post-discharge, leading to an unfortunate outcome. The prognosis for Sezary Syndrome is bleak, with a 5-year survival rate of around 28%. Elevated LDH and β2-microglobulin levels in this patient further suggested a poor prognosis, which was exacerbated by sepsis (13).

In this case, the strengths lie in its depiction of an atypical Sezary Syndrome (SS) presentation and the application of diverse diagnostic approaches (Figure 6). However, limitations exist, notably the restricted treatment options available for SS. Existing literature emphasizes well-documented prognostic factors for SS, such as advanced age, male gender, and particularly elevated level of LDH and β2-microglobulin, as predictive markers (12,13,18). Previous studies have acknowledged the rarity and aggressive nature of SS. Key take-away lessons include the importance of integrating clinical, laboratory, and histopathological findings for SS diagnosis (13). Additionally, understanding the significance of prognosis indicators like LDH and β2-microglobulin is crucial in treatment planning. When formulating treatment strategies, it's imperative to consider the patient's overall condition and potential complications. 
